# Transcriptional Sequencing and Gene Expression Analysis of Various Genes in Fruit Development of Three Different Black Pepper (*Piper nigrum* L.) Varieties

**DOI:** 10.1155/2020/1540915

**Published:** 2020-03-27

**Authors:** Choy Yuen Khew, Jennifer Ann Harikrishna, Wei Yee Wee, Ee Tiing Lau, Siaw San Hwang

**Affiliations:** ^1^Department of Research and Quality Development, Malaysian Pepper Board, Lot 1115, Jalan Utama, Pending Industrial Area, 93450 KC, Sarawak, Malaysia; ^2^School of Chemical Engineering and Science, Faculty of Engineering, Computing and Science, Swinburne University of Technology Sarawak Campus, Jalan Simpang Tiga, 93350 KC, Sarawak, Malaysia; ^3^Centre for Research in Biotechnology for Agriculture (CEBAR), Institute of Biological Sciences, Faculty of Science, University of Malaya, Kuala Lumpur, Malaysia; ^4^Monash University Malaysia, Jalan Lagoon Selatan, 47500 Bandar Sunway, Selangor Darul Ehsan, Malaysia

## Abstract

Black pepper (*Piper nigrum*) is a vital spice crop with uses ranging from culinary to pharmacological applications. However, limited genetic information has constrained the understanding of the molecular regulation of flower and fruit development in black pepper. In this study, a comparison among three different black pepper varieties, Semengok Aman (SA), Kuching (KC), and Semengok 1 (S1), with varying fruit characteristics was used to provide insight on the genetic regulation of flower and fruit development. Next-generation sequencing (NGS) technology was used to determine the flower and fruit transcriptomes by sequencing on an Illumina HiSeq 2500 platform followed by *de novo* assembly using SOAPdenovo-Trans. The high-quality assembly of 66,906 of unigenes included 64.4% of gene sequences (43,115) with similarity to one or more protein sequences from the GenBank database. Annotation with Blast2Go assigned 37,377 genes to one or more Gene Ontology terms. Of these genes, 5,874 genes were further associated with the biological pathways recorded in the KEGG database. Comparison of flower and fruit transcriptome data from the three different black pepper varieties revealed a large number of DEGs between flower and fruit of the SA variety. Gene Ontology (GO) enrichment analysis further supports functions of DEGs between flower and fruit in the categories of carbohydrate metabolic processes, embryo development, and DNA metabolic processes while the DEGs in fruit relate to biosynthetic process, secondary metabolic process, and catabolic process. The enrichment of DEGs in KEGG pathways was also investigated, and a large number of genes were found to belong to the nucleotide metabolism and carbohydrate metabolism categories. Gene expression profiling of flower formation-related genes reveals that other than regulating the flowering in black pepper, the flowering genes might also be implicated in the fruit development process. Transcriptional analysis of sugar transporter and carbohydrate metabolism genes in different fruit varieties suggested that the carbohydrate metabolism in black pepper fruit is developmentally regulated, and some genes might serve as potential genes for future crop quality improvement. Study on the piperine-related gene expression analysis suggested that lysine-derived products might present in all stages of fruit development, but the transportation was only active at the early stage of fruit development. These results indicate several candidate genes related to the development of flower and fruit in black pepper and provide a resource for future functional analysis and potentially for future crop improvement.

## 1. Introduction

Black pepper (*Piper nigrum*) is the most extensively used and traded spice crop and is often regarded as the King of Spices. Pepper usage ranges from a pure culinary spice as a condiment to use in health and medicine, since it has pharmacological benefits such as an antioxidant, in the management of type II diabetes and for treatment of hypertension [1], as an antimicrobial agent against foodborne pathogens and infectious diseases [2], as an anticancer agent reported to suppress the growth of melanoma cells [3], and as an enhancer for intestinal absorption [4]. The flavour and medicinal value of black pepper are associated with the primary and secondary metabolites produced in the fruit [5, 6]. The main component of pungency in black pepper is an alkaloid, piperine, and the unique flavour and aroma are from the essential oil constituents [7]. Despite the many and inevitable challenges faced by pepper plantations, pepper remains the world's most sought-after spice. The value of black pepper trade reached 1.5 billion US dollars from the production of 4.6 × 10^5^ tons in 2017, and 60% of the production was contributed from Southeast Asian countries (International Pepper Community Statistics).

**Figure 1:**
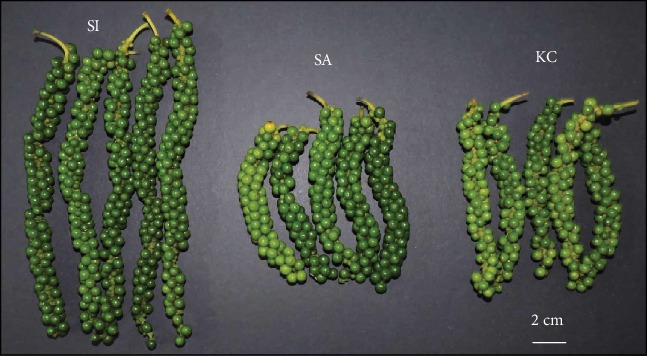
Mature fresh fruit spikes of different black pepper varieties: SI (Semongok I), SA (Semongok Aman), and KC (KC).

Black pepper fruit, botanically a drupe, contains a single seed commonly called pepper berry. In commerce, black pepper is traded as a dried whole pepper fruit, green pepper is the unripe fruit, and white pepper (whole or ground) is the ripe fruit seed. At the beginning of fruit development, the berry is pale green and soft, becoming dark green and harder as it matures. The fruit characteristics vary widely among different *P. nigrum* varieties as there is an extensive genetic variation in the black pepper germplasm [8]. Among the varieties available in Malaysia, three are commonly grown, each having some limitations in addition to the useful characteristics (Figure 1). The first is *P. nigrum* variety “Semongok Aman” (SA) which has more uniform ripening and higher secondary compound accumulation than other recommended varieties. However, it has limiting factors of shorter fruit spikes and thick fruit pericarp. The second variety is “Kuching” (KC) which has thinner pericarp, valuable for producing premium white pepper, but has poor fruit setting. The final pepper variety “Semongok I” (S1) has the most extended fruit spikes with larger berry size, but the fruit ripening is not even compared to SA (Figure 1). To unravel the regulatory processes underlying the varying phenotypes of black pepper, a systematic comparison of molecular biology data from different varieties of black pepper with distinct fruit characteristics can provide supporting data to support crop improvement.

The period for fruit development in black pepper from anthesis to fully ripened typically takes eight to nine months, depending on the growing condition and plant variety [9]. Fruit maturation and ripening vary among and within varieties as the process is a genetically programmed event and influenced by plant hormones [10, 11]. Environmental conditions during fruit development affect sugar transportation and carbohydrate metabolism mechanisms [12] and ultimately affect fruit yield and quality [13]. A recent analysis of the transcriptomes of climacteric fruit has provided valuable insights into the process of fruit development and ripening of major crops like tomato [10, 14]. However, the economically important trade spices, including black pepper, have inadequate genetic information related to flower and fruit development. Until recently, there was only one report of fruit transcriptomic data from black pepper, which emphasised the biosynthesis of piperine, black pepper's major secondary metabolite that has been associated with medicinal and health benefits [15]. The development of next-generation sequencing (NGS) platforms has provided the means for generating large sets of data to give insights into plant development through bioinformatics analysis [16]. Among the many applications, high performance *de novo* transcriptome assembly based on in-depth sequencing data provides an opportunity for genome-wide gene expression analysis of flowers and fruit [17], which is particularly useful to study black pepper flower and fruit development.

The current study is aimed at improving the understanding of the gene expression underlying molecular mechanisms of flower and fruit development in black pepper. A *de novo* transcriptome was assembled from the paired-end reads generated from sequencing twelve libraries made from the RNA of flower and fruit from three varieties of black pepper. The transcriptome data were also used to identify the genes differentially expressed between each variety as well as sequences exclusively expressed in each tissue of each variety. Gene Ontology and KEGG pathway enrichment were performed on the differentially expressed genes (DEGs) to elucidate gene functions associated with the different samples. Gene expression profiles of selected flower formation genes, sugar transport and carbohydrate metabolism genes, and piperine synthesis-related genes were further investigated from six different fruit developmental stages of the three black pepper varieties using probe-based gene expression analysis.

## 2. Materials and Methods

### 2.1. Plant Materials and RNA Preparation

Samples were collected from two-year-old mature pepper plants (*Piper nigrum* L.) of SA, S1, and KC varieties at a pepper farm in Kampong Karu, Borneo Highland Sarawak, Malaysia. Fresh flowers (1 day after anthesis, DAA) and fruit spikes (14 DAA) (Supplementary [Supplementary-material supplementary-material-1]) were collected from two biological replicates (two plants) of each pepper variety and snap-frozen in liquid nitrogen before storage at -80°C until use. Total RNA was extracted from 2 g of frozen plant tissue using a modified CTAB method [18]. The RNA was purified and treated with RQ1 RNase-free DNase (Promega, USA) to remove DNA contamination. The RNA purity and concentration were measured at 260/230 nm and 260/280 nm using a spectrophotometer (NanoPhotometer P330, IMPLEN, Germany) while the RNA integrity was measured on an Agilent 2100 Bioanalyzer with RNA 6000 Nano Reagents Kit (Agilent Technologies, 5067-1511, Lithuania). The RNA Integrity Number (RIN) was calculated using an algorithm adapted for plant RNA profiles. All RIN values were between 7.4 and 8.5.

### 2.2. Sequencing and *De Novo* Assembly

The RNA samples were sequenced as 12 libraries distributed across two lanes of an Illumina HiSeq 2500 (Illumina, USA), with 1% Illumina PhiX control library spiked into each lane at the Institute of Bioscience, University Putra Malaysia. The 100 base-pair paired-end sequencing reads were obtained in FASTQ format text file, and PhiX sequences were excluded from the raw reads. Then, the clean reads with 3′ adapter trimming were subjected to base quality (*Q* ≥ 30), ambiguous base call (*N* ≤ 2), and sequencing adapter trimming to ensure that only good quality bases derived from the mRNAs were used for the *de novo* assembly. The sequencing depth for all samples was more than 50 million reads (Supplementary [Supplementary-material supplementary-material-1]). Trimmed reads of 34 base pairs or shorter were discarded. All the assemblies were performed on a workstation with 12 cores and 64 GB random access memory using SOAPdenovo-Trans program (version 2.04; http://soap.genomics.org.cn/soapdenovo.html) and CLC Genomics Workbench (version 9.5.2). SOAPdenovo-Trans was run with parameters –I 20 –q 5 –Q 2 –H 200 –S 48 –r -F –L 200 –c 2. CLC Genomics Workbench was run with minimum-assembled-contig-length set to 200.

### 2.3. Annotation and Gene Ontology Analysis

The assembled contigs were imported in the bioinformatics tool Blast2GO (version 2.99) and were parsed on the National Center for Biotechnology Information (NCBI) of nonredundant protein database BLASTX (*E* value 1*e*-3). The functional annotation included Gene Ontology (GO) terms (http://www.geneontology.org) [19], Enzyme Commission numbers (EC code) [20], InterPro terms [21], and metabolic pathways (Kyoto Encyclopedia of Genes and Genomes (KEGG)) [22]. The “Augment Annotation by ANNEX” function was used to refine the annotations. The GOslim of the plant-specific condensed version of the GO was run for the annotation. The differentially expressed genes further underwent GO enrichment analysis by CLC Genomics Workbench (version 9.5.2) based on hypergeometric distribution [23].

### 2.4. Measurement of Gene Expression

The flower (1 DAA) and fruit tissues (14 DAA) of each variety were separately isolated for comparison purpose. Differentially expressed genes were identified using the CLC Genomics Workbench (version 9.5.2) by screening with a threshold false discovery rate (FDR) < 0.05 and log_2_ fold change (log_2_FC) > 2 or <-2. The statistical method used for the differential expression analysis in CLC Genomics Workbench is based on a negative binomial Generalized Linear Model (GLM). The use of the GLM formalism allows fitting curves to expression values without assuming that the error on the values is normally distributed. This method produced both the uncorrected *P* value and corrected FDR corrected *P* value. The number of total gene reads estimated the gene expression level located in the transcriptome based on the number of mapped reads for each unigene normalised as a Fragments Per Kb per Million reads (FPKM) value. The list of upregulated/downregulated genes was identified by filtering the differentially expressed genes via excel filter function. Once the list of upregulated/downregulated transcripts was obtained, they were compared to one another using a free web service at http://bioinformatics.psb.ugent.be/webtools/Venn/. Furthermore, a list of exclusively expressed transcripts was identified by filtering the gene expression file. The transcripts were considered uniquely expressed if they were expressed (fpkm > 0) in all replicates in one group and not expressed (fpkm = 0) in all other groups and their replicates. The GO and KEGG pathway enrichment analysis was carried out using the generated gene set from the previous analysis.

### 2.5. Probe-Based Gene Expression Analysis

The RNA for gene expression studies was prepared from the samples harvested at six developmental stages (Stage 1: 1 DAA, Stage 2: 14 DAA, Stage 3: 42 DAA, Stage 4: 175 DAA, Stage 5: 196 DAA, and Stage 6: 200 DAA) of three black pepper varieties (KC, SA, and S1) in three biological replicates (3 plants) following the CTAB method mentioned earlier. RNA samples were hybridised with gene-specific colour-coded probes for multiplexed measurement of gene expression. The NanoString Codeset was designed and synthesised by NanoString Technologies. The data retrieval was completed with the nCounter Digital Analyzer as illustrated by the manufacturer (NanoString Technologies, USA). Data was analysed and normalised using nSolver Analysis Software 3.0 (NanoString Technologies). For the quality control analysis, six positive control probes with step-wise concentrations were used to evaluate the efficiency of the hybridisation reaction and to review the linearity of the assay performance. Meanwhile, the geometric mean of eight negative probes was used to set the background threshold to control the false positive and false negative occurrences. Standard normalisation was performed using the geometric mean of six synthetic ssDNA positive control targets and three black pepper-specific housekeeping genes, ubiquinone biosynthesis protein COQ9 (COQ9), elongation factor 1-alpha (EF1a), and histone 3 (H3), to obtain the normalisation factor for the measurement of the gene expression level. The data analysis was performed with one-way ANOVA at *P* < 0.05, followed by Tukey's test to determine the significant difference in different stages.

## 3. Results

### 3.1. Transcriptome Assembly

The Illumina next-generation sequencing generated a total of 810,061,892 pair-end reads from 12 samples of flower and fruit tissues from three black pepper varieties. The sequencing platform delivered 75 GB of data output with an average read length of 93 bp. In this study, two assemblers, SOAPdenovo-Trans and CLC Workbench, were run at varying k-mer sizes of between 21 and 50 to determine the most suitable assembler and k-mer size for black pepper *de novo* assembly (assembly statistics shown in Table 1). Although the assembly from the CLC Workbench (k-mer 45) analysis provided higher coverage (about 75%) (Table 1), the total contig number was also significantly higher than that of the other assembler (113,854), suggesting that the CLC assembly had high redundancy. Thus, the SOAPdenovo-Trans assembly at k-mer size 31, with the largest maximum contig size, was used for further analysis and annotation.

Separate de novo assembly on each variety using SOAPdenovo-Trans with the same parameters allowed comparison of each of the three variety's transcriptome assemblies with the reference transcriptome using reciprocal BLAST. The reciprocal BLAST scheme returned the sequences of all contigs present in at least two varieties. The final assembly of the reference transcriptome resulted in an increase of N50 size to 1,654 bp and contig length to 12,965 bp (Supplementary [Supplementary-material supplementary-material-1]). The reference transcriptome had only a minor effect on total average length increase to 1,102 bp and only a slight decrease in contig number relative to the KC variety.

To assess the relative quality and completeness of the current assembly, we compared statistics for published *P. nigrum* fruit transcriptome [15] with the transcriptome described in this study (Table 1). The total number of sequence reads used in the current assembly represent about 810 million reads and a 14-fold increase over those used in the black pepper fruit transcriptome reported by Hu et al. [15]. The expansion of read inputs resulted in an increase of genome size from 59 million bp to 73 million bp and a larger number of unigenes. Compared with the previously published Illumina transcriptome, the inclusion of a higher number of reads had little effect on average transcript length and N50 size (Table 1). Overall, the expanded assembly represents less overall “gene space” than the previous assembly; it likely provides a more accurate reflection of the transcript landscape. Furthermore, the expanded dataset increases total coverage of transcripts relevant to tissue-specific functions.

### 3.2. Annotation and Gene Ontology Analysis

**Figure 2:**
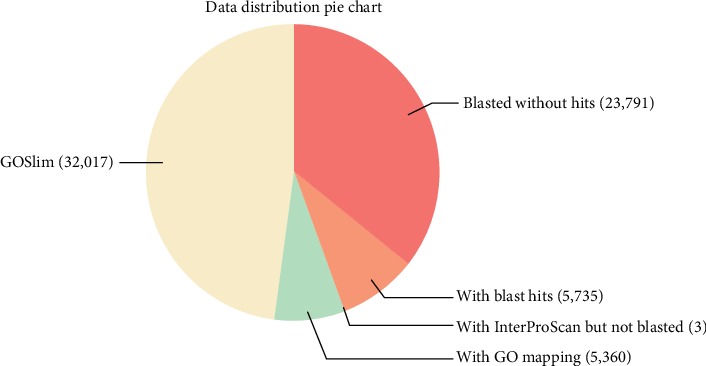
Data distribution of annotated and nonannotated sequences in black pepper flower and fruit transcriptome.

**Figure 3:**
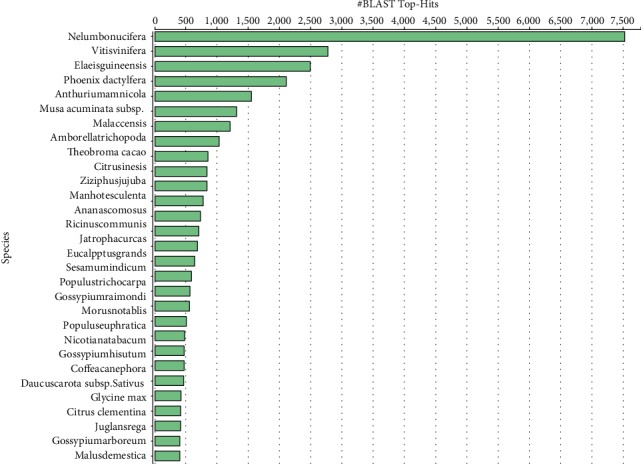
Top-Hits species distribution based on BLASTX hits in the transcriptome assembly.

The set of 66,906 assembled contigs was BLASTX parsed against the nonredundant (nr) NCBI protein database with a cut-off *E* value of 1.0^−3^, returning matches for 43,115 contigs while 23,791 had no matches (Figure 2). From the BLASTX results, *Vitis vinifera*, *Nelumbo nucifera*, *Theobroma cacao*, *Elaeis guineensis*, and *Phoenix dactylifera* were the plant species with the highest number of matches to the pepper transcriptome assembly (Figure 3). The InterProScan, Annex, and GO annotation query resulted in a 15% increase in the number of annotated sequences.

**Figure 4:**
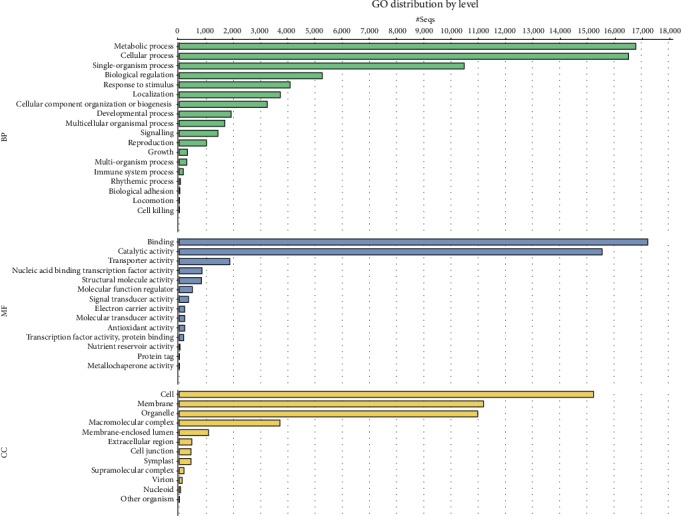
Blast2GO characterization of top 20 GO terms in the three categories of biological process, molecular function, and cellular component.

A total of 37,377 unigenes (55.8%) were assigned with at least one Gene Ontology category (Figure 4). Then, the plant-specific GO slim was used to categorize the unigenes into varying functional groups. Under the cellular component category, 46.5% of unigenes were categorized as cell, membrane, and organelle related. Meanwhile, for the molecular function category, binding and catalytic activity were the two most abundant groups. For the biological process category, the most significant proportion was under the metabolic process and cellular process (Figure 4). GO annotations showed a high number of expressed genes associated with biosynthetic processes (5,122), reproduction (556), anatomical structure morphogenesis (512), flower development (298), response to stimulus (4,112), and signalling activity (1,161). Genes involved in other important biological processes such as flower development, stress response, signal transduction, cell differentiation, pollen-pistil interaction, and fruit ripening were also identified. A total of 5,874 unigenes were mapped to 150 KEGG pathways. The pathways with the highest unigene representation were purine metabolism (map00230; 447 unigenes, 7.6%), followed by starch and sucrose metabolism (map00500; 362 unigenes, 6.2%) and phenylpropanoid biosynthesis (map00940; 241 unigenes, 4.1%).

### 3.3. Differential Gene Expression in the Transcriptome

**Figure 5:**
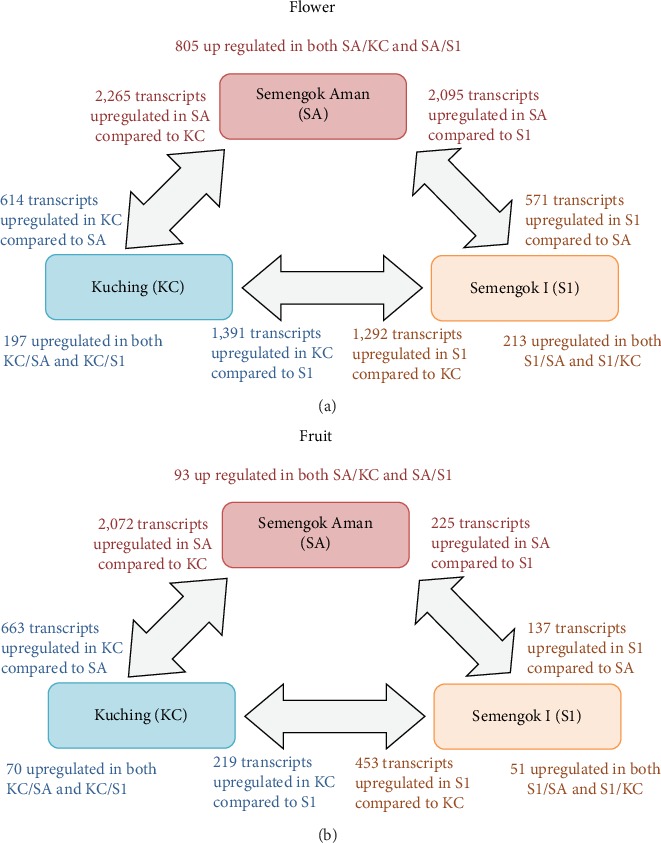
Comparison of the differential gene expression analysis of three different varieties in black pepper. Comparison of upregulated genes and common upregulated genes in the (a) flower and (b) fruit of each variety.

In this study, the FPKM method was used to calculate the expression of unigenes in black pepper flower and fruit. The transcriptome expression analysis identified a total of 66,906 unigenes, with 38,775 transcripts concordantly expressed in flower and fruit tissues of three varieties. The unigenes were considered differentially expressed when the absolute value of log_2_FC ≥ 2 and statistically significant when FDR < 0.05. From the analysis, the flower and fruit of SA show a higher number of upregulated genes when compared with KC and S1 varieties (Figure 5). There were 2,265 genes upregulated in SA flower compared with KC flower, and 2,095 genes upregulated in SA flower compared with S1 flower, of which 805 genes were found in both groups (Figure 5(a)). SA flowers had 1,519 exclusively expressed genes compared to 121 exclusively transcripts in KC and 135 unique transcripts in S1 flowers. Analysis of differentially expressed genes in the fruit of the three black pepper varieties showed SA to have the largest number of upregulated genes when compared to KC and S1 (Figure 5(b)). SA fruit also had the highest number of exclusively expressed genes, i.e., 494 genes, followed by 268 exclusively expressed genes in KC fruit and 115 exclusively expressed genes in S1 fruit.

### 3.4. Gene Ontology Enrichment Analysis

**Figure 6:**
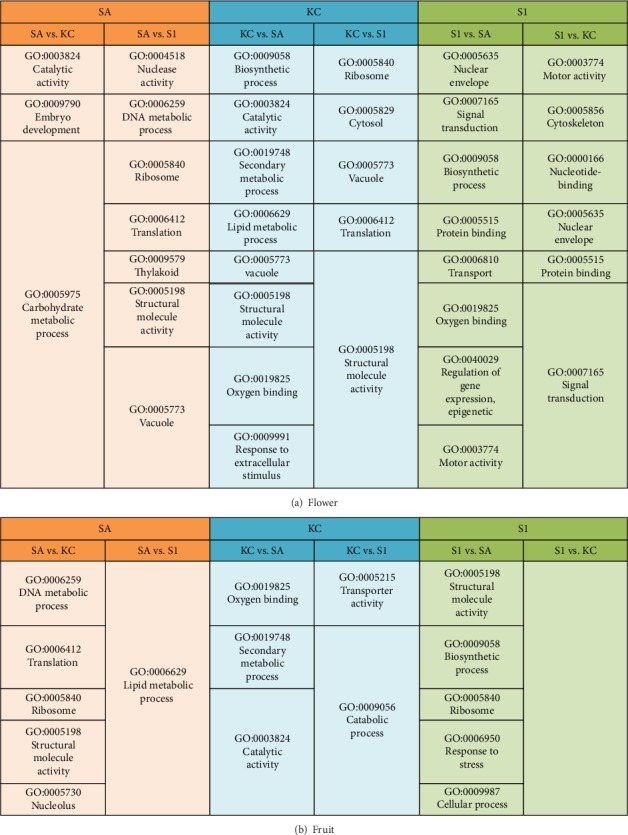
Gene Ontology (GO) term enrichment of upregulated genes in (a) flower and (b) fruit of different group comparisons in three different black pepper varieties. The GO terms in each group of comparison refer to the enriched GO terms of upregulated genes in the first variety compared to another variety. For example, the group comparison between SA and KC (SA vs. KC) is the enriched GO terms of upregulated genes in SA when compared to KC. Orange: GO terms enriched in SA. Blue: GO terms enriched in KC. Green: GO terms enriched in S1.

In order to understand the function of genes that were differentially expressed between different varieties of black pepper, GO enrichment analysis was performed on the set of upregulated genes by comparison with the transcriptome annotation in pairwise comparisons (Figure 6). Differential gene expression analysis indicated that KC has a distinct set of genes expressed when compared to SA and S1. The difference in gene expression might contribute to the smaller fruit size and shorter fruit spike in KC compared to those of the SA and S1 varieties [24]. The GO enrichment of the upregulated genes in the flower tissues of SA compared to KC indicates that the biological process GO term of carbohydrate metabolic process and embryo development was the most enriched term in SA (Figure 6(a)). These biological process GO terms coincide with the molecular function terms of catalytic activity. The functional enrichment analysis of upregulated genes in S1's flower compared to KC's flower showed specific enrichment in the molecular function terms for motor activity, coinciding with the cellular cell component of the cytoskeleton in catalysis movement along a microfilament or microtubule. Apart from this, the biological process terms of signal transduction were specifically enriched in S1 flower corresponding with the molecular function terms of protein binding and nucleotide binding to trigger a change in the activity or state of a cell. It is also noteworthy that the biological process terms of secondary metabolic process and biosynthetic process were specifically enriched in upregulated genes of KC flower when compared to SA flower. This pattern of secondary metabolite gene function was also found in the differentially expressed genes of SA fruit tissues compared to KC (Figure 6(b)). An earlier study reported that KC fruit has a higher piperine content than SA fruit [9]. The GO terms enriched in KC might be associated with the higher piperine content in the variety, so further gene expression and metabolite analysis will be useful to reveal the genetic regulation of this biosynthesis pathway. Among the GO terms that were overrepresented in the genes expressed in fruit tissues, the most significant were GO for biological processes of the DNA metabolic process and lipid metabolic process in SA, secondary metabolic process, and catabolic process in KC and biosynthetic process in S1 (Figure 6(b)). No specific GO terms were enriched in S1 fruit compared to KC fruit.

### 3.5. Expression Analysis of Flower Formation Genes

**Figure 7:**
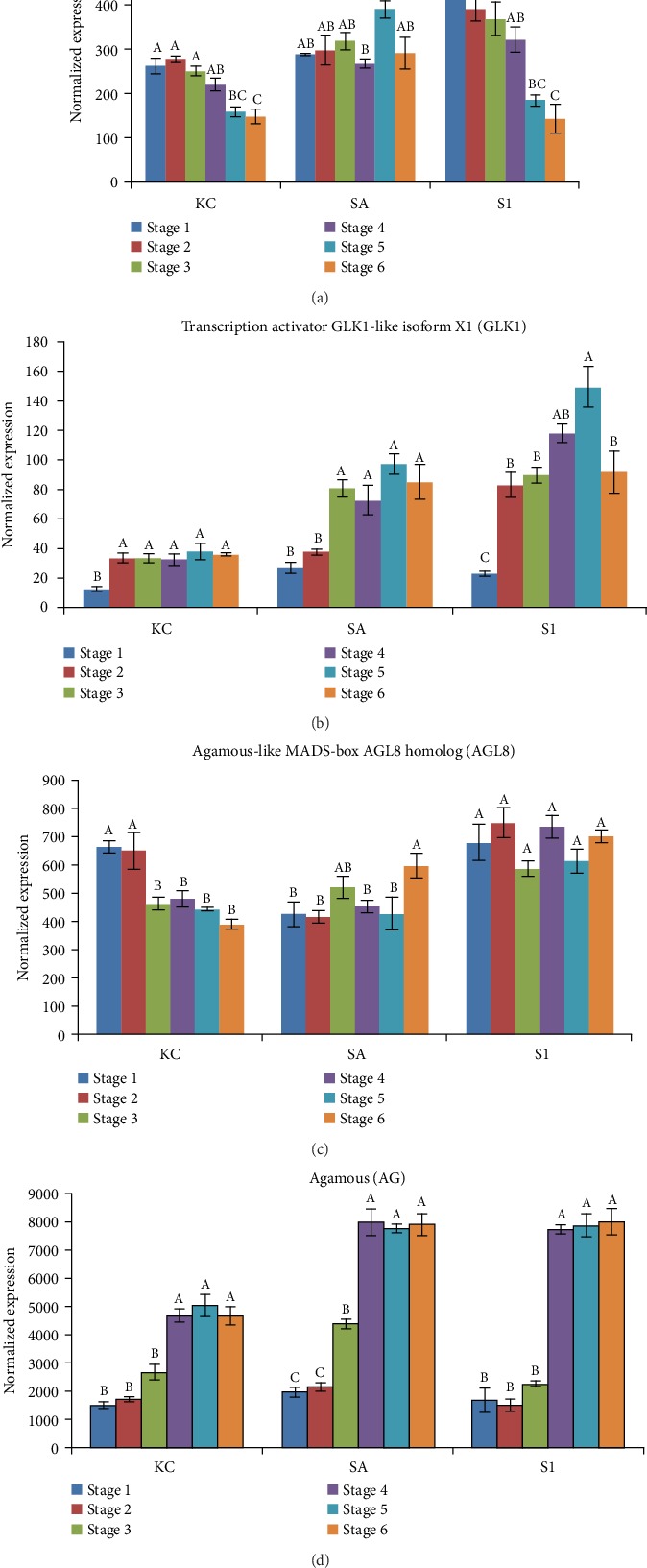
Expression profiles of six stages of fruit specific transcripts in three varieties of black pepper using probe-based gene expression analyses. (a–d) are gene expression profiles related to flower formation genes. The error bars represent the standard error from the mean expression level calculated from three biological replicates. The statistical significance analysis was based on one-way ANOVA at *P* < 0.05. Means followed by the same letter within stages of each variety are not significantly different by Tukey's test at *P* < 0.05.

Four genes related to flower formation that exhibited high fold changes in differential expression analysis were further analysed at six different fruit development stages (Figure 7). Overall, the three different black pepper varieties have distinct gene expression profiles throughout the fruit development stages. KC and S1 varieties exhibited high expression of *transcription factor APETALA2* (*AP2*) at the flower stage (Stage 1) and expression decreased as fruit matured. The expression of Agamous-like MADS-box AGL8 homolog (AGL8) remained high across the fruit development stages in SA and S1 varieties but slightly declined in the KC variety. Transcription activator GLK1-like isoform X1 (GLK1) and Agamous (AG) were significantly highly expressed at the late stage of fruit development in most of the varieties except that the expression of GLK1 was levelled off across fruit development stages in KC variety. All the gene expression analysis throughout this study was normalised against the housekeeping genes of ubiquinone biosynthesis protein (COQ9), histone 3 (H3), and elongation factor 1-alpha (EF1a) with no significant difference across fruit development stages in all three varieties (Supplementary [Supplementary-material supplementary-material-1]). The transcript counts of housekeeping genes were normalised using six synthetic ssDNA positive control targets.

### 3.6. Expression Analysis of Genes Related to Sugar Transporter and Carbohydrate Metabolism

**Figure 8:**
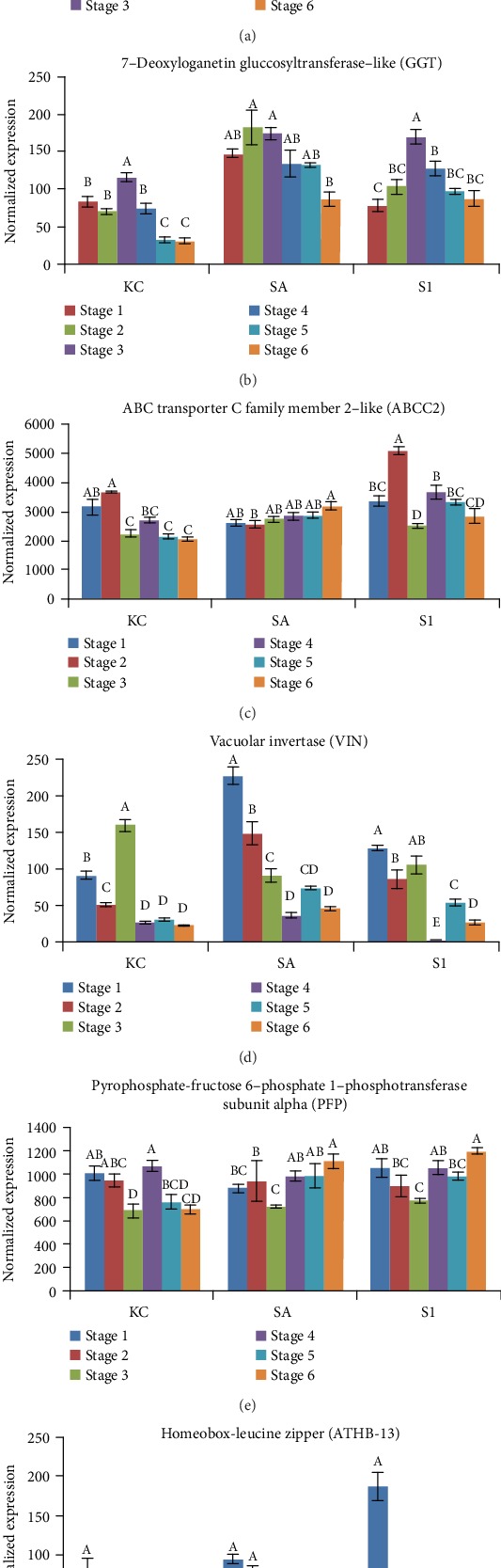
Expression profiles of genes related to sugar transporter and carbohydrate metabolism in six stages of fruit development in three varieties of black pepper using probe-based gene expression analyses (a–f). The error bars represent the standard error from the mean expression level calculated from three biological replicates. The statistical significance analysis was based on one-way ANOVA at *P* < 0.05. Means followed by the same letter within stages of each variety are not significantly different by Tukey's test at *P* < 0.05.

Sugar transporter genes and carbohydrate metabolism genes are essential to fruit development. Six differentially expressed genes related to sugar transport and carbohydrate metabolism were identified in the transcriptome based on their log_2_ fold change and *P* value (Figure 8). Each pepper variety produced a distinct gene expression profile. The profiles for sugar transporter ERD6-like 16 (ERD6) showed a general decrease in expression as the fruit ripened in KC variety; however, this was not shown in the SA and S1 varieties. The gene expression of 7-deoxyloganetin glucosyltransferase-like (GGT) peaked at the fruit setting stage, and the homeobox-leucine zipper (ATHB-13) gene was only highly expressed at the early stage of fruit development in all three varieties. There were significant differences in gene expression at some stages for each of the varieties for the ABC transporter C family member 2-like (ABCC2), vacuolar invertase (VIN), and pyrophosphate-fructose-6-phosphate 1-phosphotransferase subunit alpha (PFP). At the same time, there were no significant differences across the fruit development stages for the housekeeping genes.

### 3.7. Expression Analysis of Secondary Metabolite-Related Genes for the Biosynthesis of Piperine

**Figure 9:**
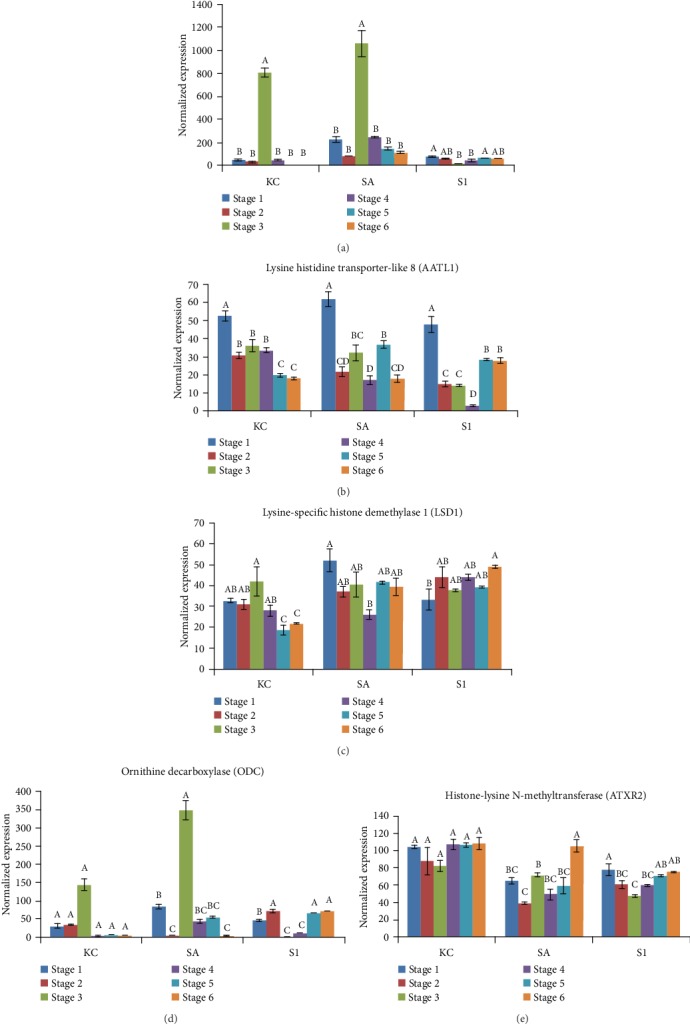
Expression profiles of secondary metabolite-related genes for the biosynthesis of piperine in six stages of fruit development in three varieties of black pepper using probe-based gene expression analyses (a–e). The error bars represent the standard error from the mean expression level calculated from three biological replicates. The statistical significance analysis was based on one-way ANOVA at *P* < 0.05. Means followed by the same letter within stages of each variety are not significantly different by Tukey's test at *P* < 0.05.

In this study, five genes that are involved in secondary metabolite biosynthesis pathways revealed from the transcriptome were further analysed at different fruit development stages of the three different black pepper varieties (Figure 9). The five genes selected are homologs of genes involved in lysine/ornithine metabolism, related to piperine biosynthesis [15, 25]. Significant high expression of (-)-isopiperitenol (-)-carveol dehydrogenase (ISPD) and Ornithine decarboxylase (ODC) was observed during the fruit expansion stage in KC and SA varieties compared to low expression of ISPD and ODC across stages in S1 varieties. Meanwhile, the lysine histidine transporter-like 8 (AATL1) was significantly highly expressed at the flower stage (Stage 1) in all three varieties but had a decreased level of transcript as the fruit ripened. Similar levels of expression of lysine-specific histone demethylase 1 (LSD 1) and histone-lysine N-methyltransferase (ATXR2) were observed across the stages in all three varieties while some stages showed a significant difference in the expression levels.

## 4. Discussion

### 4.1. The Fruit and Flowers of Different Varieties of Black Pepper Have Several Overlapping but Also Uniquely Expressed Transcripts

The present study provides the first flower and fruit transcriptomic view of different varieties of *P. nigrum*. The *de novo* assembly provides useful genetic information and the identification of genes and gene categories expressed in flower and during fruit development in black pepper. The transcriptome not only influenced the data coverage but also changed the number of gene sequences available for downstream applications such as the study of tissue-specific gene expression [26]. Based on paired-end sequencing of RNA-Seq libraries prepared from mRNA isolated from flower and fruit tissues, a SOAPdenovo-Trans assembly and a CLC Workbench assembly were compared. Here, we demonstrated that SOAPdenovo-Trans produced a higher quality *de novo* assembly of the black pepper flower and fruit transcriptome. The relative superiority of SOAPdenovo-Trans to CLC Workbench was reported earlier [27, 28]. SOAPdenovo-Trans includes the feature of an error-removal model from Trinity and the robust heuristic graph traversal approach from Oases. Moreover, SOAPdenovo-Trans simplifies the scaffolding graphs through a strict transitive reduction method [29]. Previous studies have shown that SOAPdenovo-Trans provided higher contiguity, lower redundancy, and faster execution [30, 31]. Our current assembly of a *P. nigrum* transcriptome compared favourably with transcriptomes produced with NGS in previous studies. The average length of the *P. nigrum* assembly was 1,102 bp (Table 1) which was higher than the 449 bp reported for *P. nigrum* cv. Panniyur 1 [8]. The N50 length was similar to that reported from the assembly by Hu et al. [26] of the fruit transcriptome from *P. nigrum* cv. Reyin 1.

Gene annotation is important to understand the biological function within NGS data and for comparison with data from other studies and species. In our case, BLAST2GO with comprehensive functional annotation for this nonmodel plant was used for gene annotation. Among all the databases, including KEGG, GO, Inter-Pro, and NR, annotated unigenes accounted for 64.44% of the total number of unigenes in the current black pepper transcriptome. Considering the shortage of sequence data for *P. nigrum*, the level of annotation seems reasonable, similar to values reported previously, i.e., 65.43% [15]. As in reports for other fruit plants, an enormous number of genes (24,011) were annotated with categories related to binding and catalytic activity [15, 17]. About 2,400 transcripts were annotated with “DNA binding,” and from it, around 800 were further annotated precisely with GO term “sequence-specific DNA binding transcription factor activity.” This result possibly shows that the sequences might represent the expressed transcription factors involved in the regulation of gene expression during flower and fruit development. Furthermore, the *P. nigrum* flower and fruit transcriptome were annotated to encode proteins associated with terms related to biosynthetic process, reproduction, anatomical structure morphogenesis, flower development, and secondary metabolic process, showcasing the active processes happening in the developing fruit. Therefore, GO annotation identified a broad set of candidate genes which are believed to be involved in the control of fruit development and the bioactive secondary metabolite synthesis.

A differential gene expression dataset was established to elucidate the molecular mechanisms and regulatory pathways of flower and fruit in black pepper through the comparison among three different varieties. From the analysis, the KC variety of black pepper was found to have a distinct set of genes expressed in flower and fruit compared to that of the SA and S1 varieties. A majority of these DEGs were represented under the biological process terms of carbohydrate metabolic process, embryo development, and DNA metabolic process. These biological processes are the primary metabolisms in flower tissues and interact with each other for the growth and development of the flower. Several previous studies have presented the involvement of these pathways in flower development, such as pollen development, stamen development, floral opening, and senescence [32–34]. The genes regulating the secondary metabolic process and response to extracellular stimulus were observed as upregulated in KC flower and fruit when compared to their expression levels in SA flower and fruit. This result supports the synthesis of secondary metabolites such as flavonoids and alkaloids during the reproduction process and is consistent with the higher piperine content reported for KC compared to SA and S1 [9]. Interestingly, as plant hormones have been previously shown to be necessary to signal the flower formation, fruit set, and fruit maturation in black pepper [24, 35], the high numbers of genes in the category of signal transduction and cell-cell signalling in S1 are consistent with the importance of expression of small molecule like plant hormones during fruit development [36]. Therefore, it would be useful to further study these groups of genes, related to growth and to secondary metabolism for future crop improvement.

### 4.2. Flower Formation Genes Control Flowering Time and Contribute to Fruit Development and Ripening

Black pepper production is hampered by the nonsynchronous nature of flowering and uneven fruit ripening which reduces the fruit quality in black pepper. Therefore, it is critical to explore the molecular mechanism underlying flower formation for better understanding and application to the management of flowering time to increase black pepper productivity and quality. MADS-box gene families are important regulatory factors to control flower transition in plants [37]. The Agamous-like MADS-box AGL8 homolog had been reported from earlier studies in the model plant *Arabidopsis* to regulate the establishment of carpels during fruit development [38] and also regulating the flowering time [39]. AGL8 is implicated in the network of flowering time control by directly binding and being activated by AP2, which is both the flowering repressor and A-class flower identity gene [38]. In this study, the expression profiles of the pepper homologs of AP2 and AGL8 also implicated their role in flowering with their high expression at the flowering stage with complete emergence of stigma in all three varieties (Figures 7(a) and 7(c)). It is noteworthy that despite the hypothesised role, the AGL8 was also highly expressed at the late stage of fruit development when the AP2 transcript was low. This result suggests that other than acting as a flower controlling gene, AGL8 may also contribute to fruit development in black pepper. The pepper homolog of another C-type MADS-box gene, Agamous (AG), demonstrated high expression at the late stage of fruit development in all three varieties (Figure 7(d)) which supports the results from AGL8 as well as a previous study in grapevine [40] that AG has a regulatory role in flower development and also acts as a key regulator in fruit development in black pepper.

Golden two-like (GLK) family members are involved in coordinating the development and maintenance of chloroplasts [41]. Study of the transcription activator of the GLK1 black pepper homolog in this study showed low transcript levels at the flowering stage, which suggests that GLK may act as a negative regulator of flowering. According to Zubo et al. [41], elevated expression of GLK will delay the flowering time in *Arabidopsis*. However, another study has reported that high expression of GLK in tomato enhances the fruit photosynthesis rate and chloroplast development, which contribute to increased carbohydrates and carotenoids in the fruit, leading to uniform fruit ripening [42]. Therefore, increase in expression of GLK1 toward the fruit ripening stage in SA and S1 varieties in black pepper (Figure 7(b)) could imply its role in the fruit development and ripening activity in black pepper.

### 4.3. Sugar Metabolism and Transporter Genes Regulating Fruit Size, Secondary Metabolite Formation, Flowering Time, and Pollen Germination in Black Pepper

Sucrose metabolism is essential to fuel plant processes including signalling, yield formation and nucleic acid synthesis, and transport to the sinks through the predominant sucrose-controlled phloem transport pathway [43]. As the first product of photosynthesis, sugar is translocated from the photosynthetic part to nonphotosynthetic sinks, including reproductive organs (i.e., flower) and fruit in plants [44]. Analysis of ERD6, a sugar transporter gene, showed contrasting patterns of expression among the three different black pepper varieties across the fruit developmental stages (Figure 8(a)). The pattern of decreasing expression through the fruit developmental stages in KC variety (and the high expression of ERD6 over the fruit developmental stages in SA and S1 varieties) is believed to be connected with the sugar content level in the variety and might be associated with the different fruit size of each variety especially for KC variety with comparatively smaller fruit size (0.62 cm) than SA (0.68 cm) and S1 (0.67 cm) (Figure 1) [24]. This finding suggests ERD6 as a candidate gene for future study on the sugar content regulation in black pepper by experimenting on functional gene study to manipulate the gene expression to evaluate the gene function further.

Next, the analysis of 7-deoxyloganetin glucosyltransferase-like (GGT) gene in this study revealed high expression of GGT in SA compared to KC and S1 (Figure 8(b)). Glucosyltransferases have been reported earlier to be linked to secondary metabolite metabolism by catalysing carbohydrate moieties into natural compounds [45, 46]. SA variety has a higher chemical content of volatile and nonvolatile oils than other varieties, and therefore, the active response of GGT in SA might be linked to the formation of secondary metabolites in the variety. Further study of the GGT impaired mutant plants may provide more understanding of the role of GGT in the synthesis of secondary metabolites.

Sucrose metabolism is also a critical step in reproductive organ formation, and a previous study has reported that silencing the vacuolar invertase (VIN) gene in cotton led to a failure in pollination [44]. In this study, VIN was highly expressed at the flowering and early fruit development stages, suggesting a role of VIN in the black pepper flower development process (Figure 8(d)). A study reported by Heyer et al. [47] also showed that flowering time control is strongly affected by the different levels of sugar in the apex, and VIN play a crucial role in this network. VIN coordinating carbon dioxide uptake by sugar-mediated signalling pathways and influencing apex sugar levels [48]. High expression of VIN in SA variety that exhibited a more uniform ripening characteristic suggests a role for VIN in controlling the flowering time, i.e., inducing the transition to flower, and it may be a potential gene for manipulating the synchronisation of flowers in black pepper. Improving the flowering trait in black pepper could be achieved through targeted VIN gene overexpression in the plants.

The black pepper pyrophosphate fructose-6-phosphate 1-phosphotransferase (PFP) was ubiquitously expressed in various stages of fruit development in all three different black pepper varieties (Figure 8(e)). This result is in agreement with previous research in rice as PFP is expressed in both early and late stages of grain filling, modulating carbon metabolism [49]. Another gene which has an active role in the sucrose signalling pathway, homeobox leucine zipper (ATHB-13), is a transcription factor [50] that was highly expressed only at the early stage of fruit development in all three black pepper varieties (Figure 8(f)). High expression of ATHB-13 at the flowering stage of black pepper may have an association with pollen germination as has been reported for *Arabidopsis* [51]. ATHB-13 regulates pollen germination by modifying the expression of pollen coat genes and genes involved in cell development and organisation as well as protein and lipid transportation [52, 53].

### 4.4. Expression of the Piperine-Related Genes Was Associated with the Biosynthesis of Secondary Metabolites in Different Varieties

Black pepper is valued for the presence of piperine that gives the flavour of pungency [54]. Piperine (1-piperoylpiperidine) belongs to the most important group of nitrogenous secondary metabolites referred to as alkaloids [15]. Study of the biosynthesis of piperine has shown that piperine is derived from the primary metabolism of lysine/ornithine in the *Punica granatum*, a Piper species [55]. However, information on the molecular mechanism on piperine biosynthesis in black pepper is still limited. In this study, the unigenes of homologs involved in the lysine/ornithine metabolism-related pathway were manually identified from the transcriptome. Among them, five genes were further profiled in six different fruit development stages in three different varieties (Figure 9). The lysine-specific transcripts, LSD1 and ATXR2, were found to be expressed throughout the fruit development stages (Figures 9(c) and 9(e)). Both genes are involved in the biosynthesis of piperine from lysine [56]. The expression profiles from LSD1 and ATXR2 suggest that the biosynthesis of the lysine-derived alkaloid is active across the fruit developmental stages, though quantification of piperine across the fruit developmental stages will be needed to validate the levels of these secondary metabolites.

AATL1 facilitates lysine and histidine transportation across the cellular membrane in higher plants [57]. AATL1 expression declined from early fruit development to fruit ripening in all three varieties of black pepper (Figure 9(b)). The reason for the decline in expression of AATL1 as the fruit ripened is not completely clear. Generally, it is assumed that more active transportation of lysine products will occur at the early stage of fruit development but become constrained as fruit matures and ripens. This assumption is based on a study in pear fruit that some transporter genes including AATL1 were involved in the early stages of fruit cell expansion for the generation of high osmotic stress in vacuoles of young fruits. In contrast, some transporter genes are expressed more highly at late stages of fruit development for the accumulation of sugar and organic acid for fruit maturation and dispersal [58]. The expression of ISPD and ODC increased markedly at the fruit expansion stage in KC and SA varieties but not in the S1 variety (Figures 9(a) and 9(d)). Previous studies confirmed that ISPD was important to catalyse the oxidation of isopiperitenol [59], whereas ODC is the enzyme that catalyses the conversion of ornithine to polyamine [60, 61]. Both genes are involved in the biosynthesis of aromatic compounds [62], and the gene expression profiles in KC and SA varieties (Figures 9(a) and 9(d)) might be associated with the higher concentration of aromatic compounds synthesised at the fruit expansion stage in these two varieties compared to those in the S1 variety. Overall, more genetic functional characterisation work is needed to positively determine the functionality and specificity of the genes in the fruit development of black pepper.

## 5. Conclusion

In this study, the assembled transcriptome of black pepper flower and fruit has presented a global description of expressed genes in black pepper fruit development. Identification of these genes provides a rich source for further dissection of the molecular mechanisms that govern black pepper fruit development and ripening. The GO enrichment revealed many genes involved in purine metabolism, starch and sucrose metabolism, signal transduction, and secondary metabolite biosynthesis. The current findings indicated that AP2 and AGL8 might be significant regulators of flowering in black pepper, while GLK1 and AG might be necessary for fruit development as well as to synchronise flowering. The comparison of differentially expressed genes related to sugar transport and carbohydrate metabolism in three different black pepper varieties (SA, KC, and S1) further provides a relationship between the differential gene expression and the different morphological characteristics of the black pepper varieties. The LSD1 and ATXR2 gene expression profiles suggest that lysine-derived alkaloids might be present in all stages of fruit development, but the transportation of lysine products may only be active at an early stage of fruit development as shown in the AATL1 gene expression data. The current study is the first insight into the genetic mechanism of flower and fruit development in black pepper of different varieties. Overall, it is interesting to study the function of genes identified from the differential gene expression analysis and represented in Gene Ontology and pathway enrichment to understand the more detail on the flower and fruit development in black pepper. Nevertheless, further functional gene analysis and metabolite profiling will be valuable for targeted crop improvement of black pepper.

## Figures and Tables

**Table 1:** Summary of transcriptome data from two assemblers after redundancy removal and a previously published black pepper fruit transcriptome.

Assembler	Soap-Trans				CLC	Trinity^a^
k-mer	21	23	27	31	45	—
N50 size	1,641	1,639	1,653	1,654	1,020	1,757
N50 no.	13,546	14,074	14,587	14,809	26,118	—
Unigene number	61,006	63,669	66,025	66,906	113,854	44,061
Genome size	67,404,744	70,065,254	72,708,828	73,742,859	93,269,479	59,262,045
Average length	1,105	1,100	1,101	1,102	819	1,345
Min contig	200	200	200	200	295	300
Max contig	9,932	9,884	9,781	12,965	12,375	15,000
Mapping				72%	75%	—

Note: the assembly statistics are from two assemblers after the removal of redundant contigs from the initial assembly. ^a^Data from Hu et al. [15].

## Data Availability

The datasets used and generated during this study are included in this published article. The transcriptome data that support the findings of this study are available from the website of Malaysian Pepper Board under Malaysian Pepper Transcriptome Sequencing. The data can be accessed through the link of http://mpts.mpb.gov.my/mpgd/login_user.php.

## Supplementary Materials

Supplementary Materials Supplementary material 1: flower and fruit samples of *P. nigrum*. Completely emerged flower 1 DAA of A: SA, B: S1, and C: KC. Fruit half covered by subtracting bracts (14 DAA) in D: SA, E: S1, and F: KC. Supplementary material 2: read coverage for flower and fruit samples of *P. nigrum*. Supplementary material 3: summary of de novo assembly of transcriptome from each variety. Supplementary material 4: all the gene expression analysis throughout this study was normalised against the housekeeping genes of ubiquinone biosynthesis protein (PnCOQ9) (Panel A), histone 3 (PnH3) (Panel B), and elongation factor 1-alpha (PnEF1a) (Panel C) with no significant difference across fruit development stages in all three varieties. The transcript counts of housekeeping genes were normalised using six synthetic ssDNA positive control targets.
