# Supplementary material for: Transcriptional Sequencing and Gene Expression Analysis of Various Genes in Fruit Development of Three Different Black Pepper (Piper nigrum L.) Varieties
Source: Int J Genomics. 2020 Mar 27;2020:1540915. doi: 10.1155/2020/1540915 (PMC7210556; doi:10.1155/2020/1540915)

**Electronic supplementary materials**

**Title:**

Transcriptional sequencing and gene expression analysis of various genes in fruit development of three different black pepper (*Piper nigrum* L.) varieties.

**Authors:**

Choy Yuen Khew, Harikrishna Jennifer Ann, Wei Yee Wee, Ee Tiing Lau and Siaw San Hwang,

**Journal:**

International Journal of Genomics

**Corresponding author:**

Choy Yuen Khew

Address: Department of Research and Development, Malaysia Pepper Board, Lot 1115, Jalan Utama, Pending Industrial Area, 93450 Kuching, Sarawak, Malaysia

Tel: +6082-331811(141)

Fax: +6082-336877

E-mail: cykhew@mpb.gov.my

**Content:**

**Supplementary material 1**

**Supplementary material 2**

**Supplementary material 3**

**Supplementary material 4**

**Supplementary material 1:** Flower and fruit samples of *P. nigrum*. Completely emerged flower 1 DAA of A: SA; B: S1; C: KC. Fruit half covered by subtracting bracts (14 DAA) in D: SA; E: S1; F: KC.


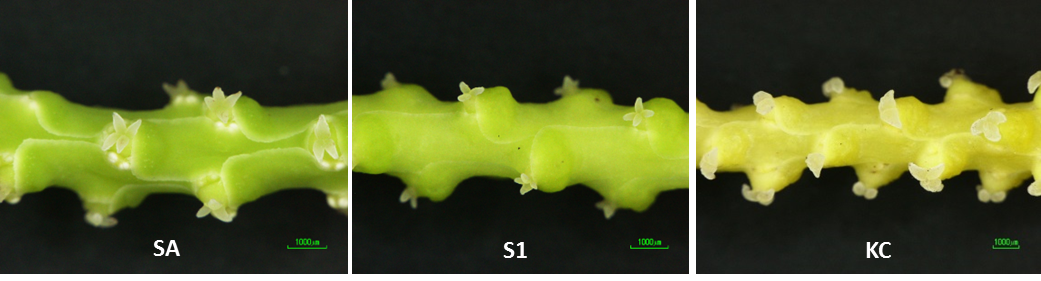

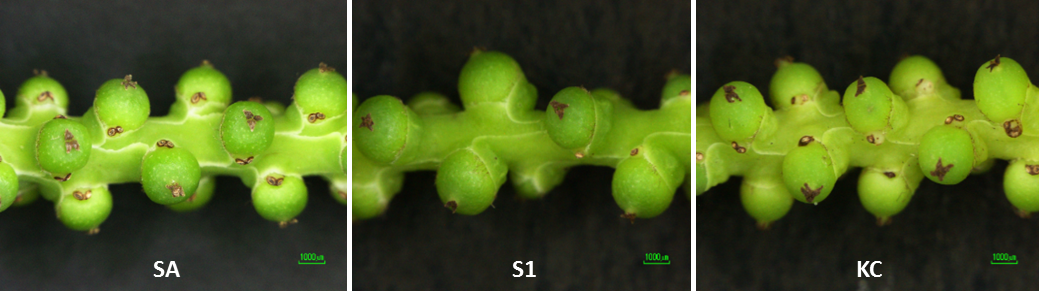


**A**

**C**

**B**

**D**

**E**

**F**

1000µm

1000µm

1000µm

1000µm

1000µm

1000µm

**Supplementary material 2:** Read coverage for flower and fruit samples of *P. nigrum*.


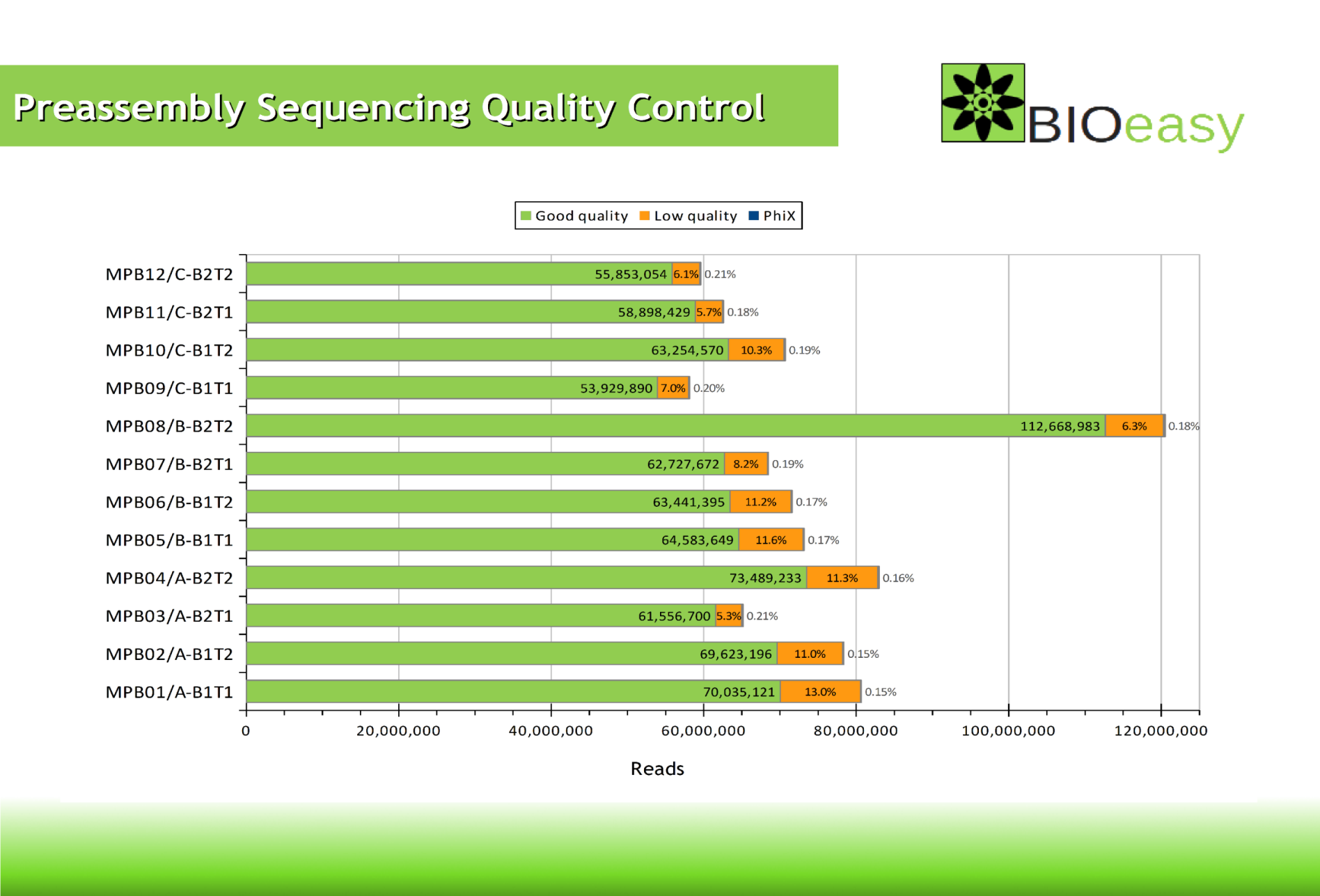


**S1 Fruit 2**

**SA Fruit 2**

**SA Flower 2**

**SA Fruit 1**

**SA Flower 1**

**KC Fruit 2**

**KC Flower 2**

**KC Fruit 1**

**KC Flower 1**

**S1 Flower 2**

**S1 Fruit 1**

**S1 Flower 1**

**Supplementary material 3:** Summary of de novo assembly of transcriptome from each variety.

| **SOAP-denovo** | **SA** | **KC** | **S1** | **Final Assembly** |
| --- | --- | --- | --- | --- |
| **N50 size** | 1418 | 1399 | 1508 | 1654 |
| **N50 no** | 16170 | 17306 | 14560 | 14809 |
| **Contig number** | 72260 | 85393 | 67978 | 82224 |
| **Transcriptome size** | 71820603 | 77381252 | 67349922 | 73742859 |
| **Average length** | 993.919222 | 906.1779303 | 990.76057 | 1102 |
| **Min contig** | 200 | 200 | 200 | 200 |
| **Max contig** | 8590 | 10350 | 10732 | 12965 |

**Supplementary material 4:** All the gene expression analysis throughout this study was normalised against the housekeeping genes of *ubiquinone biosynthesis protein* (*PnCOQ9*) (Panel A), *histone 3* (*PnH3*) (Panel B) and *elongation factor 1-alpha* (*PnEF1a*) (Panel C) with no significant difference across fruit development stages in all three varieties. The transcript counts of housekeeping genes were normalised using six synthetic ssDNA positive control targets.


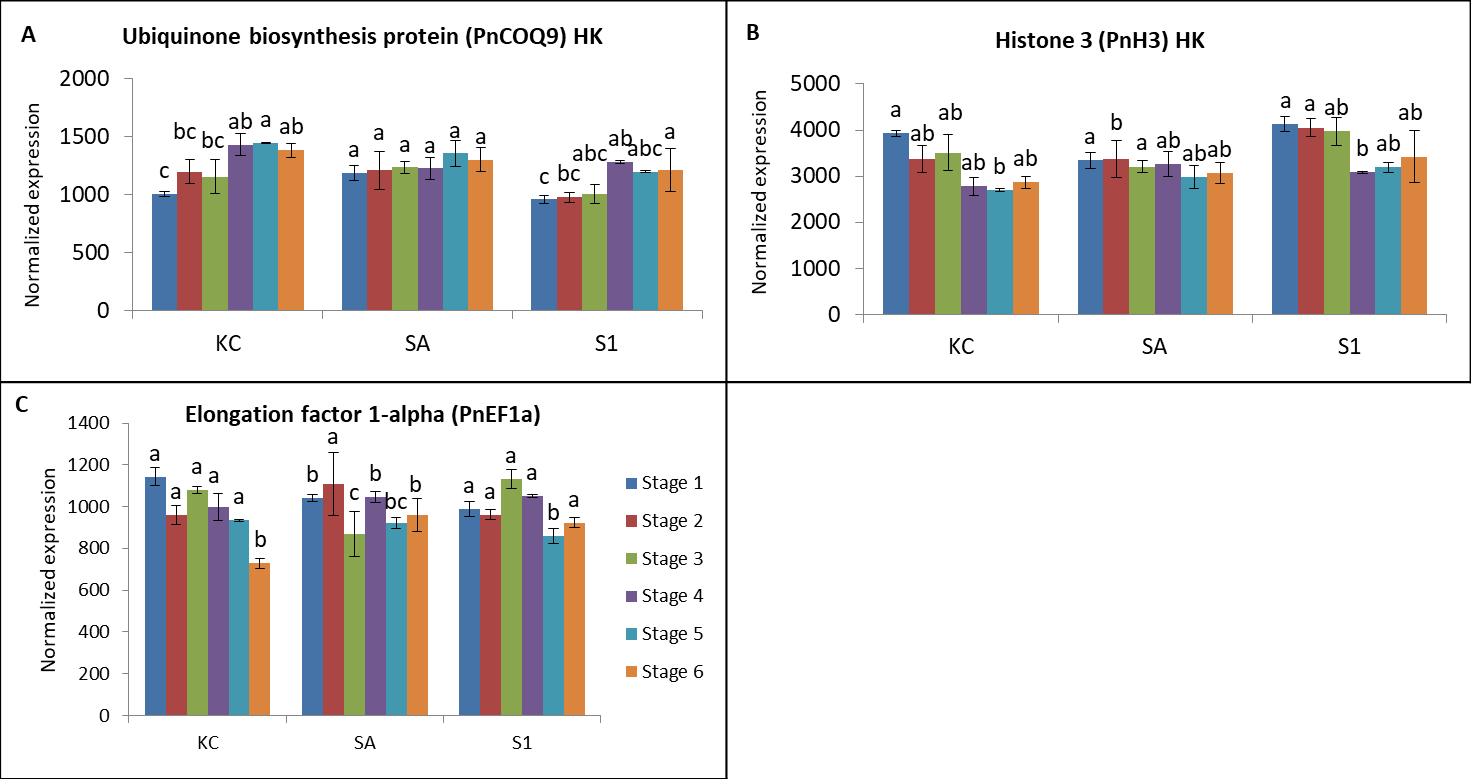

Supplement: Supplementary Materials — Supplementary material 1: flower and fruit samples of P. nigrum. Completely emerged flower 1 DAA of A: SA, B: S1, and C: KC. Fruit half covered by subtracting bracts (14 DAA) in D: SA, E: S1, and F: KC. Supplementary material 2: read coverage for flower and fruit samples of P. nigrum. Supplementary material 3: summary of de novo assembly of transcriptome from each variety. Supplementary material 4: all the gene expression analysis throughout this study was normalised against the housekeeping genes of ubiquinone biosynthesis protein (PnCOQ9) (Panel A), histone 3 (PnH3) (Panel B), and elongation factor 1-alpha (PnEF1a) (Panel C) with no significant difference across fruit development stages in all three varieties. The transcript counts of housekeeping genes were normalised using six synthetic ssDNA positive control targets. [file 1540915.f1.docx]
